# A semi-systematic review on hypertension and dyslipidemia care in Egypt—highlighting evidence gaps and recommendations for better patient outcomes

**DOI:** 10.1186/s42506-021-00096-9

**Published:** 2021-12-01

**Authors:** Ashraf Reda, Hany Ragy, Kanwal Saeed, Mohammed Ashraf Alhussaini

**Affiliations:** 1 Department of Cardiology, Menoufia University, Shebin El Kom, Egypt; 2 Department of Cardiology, National Heart Institute, Cairo, Egypt; 3 Legacy employee, Research, Development and Medical, Pfizer Upjohn, Dubai, United Arab Emirates; 4 Medical Affairs, Viatris, Cairo, Egypt

**Keywords:** Cardiovascular disease, Dyslipidemia, Egypt, Hypertension, Patient-centric, Prevalence, Touchpoints

## Abstract

**Background:**

Both hypertension and dyslipidemia are considered as major modifiable risk factors of cardiovascular diseases (CVDs), and their prevalence in Egypt has increased in recent years. Evidence-based systematic evaluation of data on hypertension and dyslipidemia is critical for effective patient-centric management to reduce the overall risk of CVDs in Egypt. This semi-systematic review aimed to quantify and identify data gaps in the prevalence and distribution of patient journey touchpoints including awareness, screening, diagnosis, treatment, adherence, and control of hypertension and dyslipidemia to provide the basis for research prioritization, practice guidance, and health care reforms in Egypt.

**Main body:**

Structured search was conducted on MEDLINE and Embase to identify articles published in English between January 2010 and December 2019 that reported key patient journey touchpoints in hypertension and dyslipidemia management. Unstructured search was conducted on public or government websites with no date restriction. Data from all sources were extracted and presented descriptively. In total, 22 studies published between 1995 and 2020 on hypertension and dyslipidemia were included in the final analyses. The prevalence of hypertension in Egypt ranged from 12.1 to 59%. Studies reported awareness (37.5% and 43.9%), diagnosis (42% and 64.7%), treatment (24% and 54.1%), and adherence to antihypertensive medication (51.9%) to be low. Furthermore, the percentage of patients who had their blood pressure controlled ranged from 8 to 53.2%. The prevalence of dyslipidemia varied in the general population (range 19.2–36.8%) but was higher in patients with acute coronary syndrome (ACS) (50.9% and 52.5%) and coronary artery disease (58.7%). A national report indicated that 8.6% of the general population was screened for dyslipidemia; however, no data was available on the diagnosis and treatment rates. Among ACS patients, 73.9% were treated for dyslipidemia. Data indicated low levels of medication adherence (59%) among dyslipidemia patients, with overall low control rates ranging from 5.1 to 34.4% depending on CVD risk in populations including ACS patients.

**Conclusion:**

Data on patient journey touchpoints of hypertension and dyslipidemia are limited in Egypt, indicating the need for more systematic and high-quality evidence-based studies covering different aspects of patient-centric management for better management of CVD and its risk factors.

**Supplementary Information:**

The online version contains supplementary material available at 10.1186/s42506-021-00096-9.

## Background

Noncommunicable diseases (NCDs) were responsible for 42 million deaths worldwide in 2019, with a majority of these deaths (18.6 million) attributed to cardiovascular diseases (CVDs) [1]. There is an increasing focus on reducing mortality due to NCDs by 2030, as proposed by the World Health Organization (WHO) under the Sustainable Development Goals [2]. Despite these efforts, there was an increase in CVD mortality rate in the low- and middle-income countries (LMICs) between 2000 and 2012 [3], which contributed to 80% of global CVD deaths [4].

Egypt, classified as an LMIC by the World Bank, reported an annual mortality rate of 40% due to CVDs. Being the most populous country in the Middle East and North African region, Egypt accounted for 15% of the CVD mortality for the entire region [5–7]. The high CVD mortality in Egypt was attributed to increasing urbanization in the country, accompanied by widening gaps in the socio-economic statuses, increasing westernization of lifestyles, and rising imbalance between improved health services and accessibility to the population [3, 8–12]. These changes explained the rise in the prevalence and possible complications in the management of modifiable and preventable CVD risk factors such as hypertension and dyslipidemia [13]. The most recent global estimates suggested that hypertension affected 1.13 billion people worldwide [14]. According to the WHO global estimates, the prevalence of dyslipidemia among adults is also quite high at 39% [15]. In Egypt, a few population-based studies have estimated the prevalence of CVD risk factors; the most recent national survey reported the prevalence of hypertension and dyslipidemia at 29.5% and 19.2%, respectively [16].

The management of hypertension in Egypt was affected by multiple factors such as an inconsistent approach adopted by the physicians to measure blood pressure, inadequate treatment, lower number of patients achieving target blood pressure, lower awareness of hypertension among patients, limited use of risk assessment tools to identify high-risk patients, patient noncompliance to treatment, lack of preventive centers, and absence of national registries for CVD risk factors [5, 17–20]. On the other hand, despite higher awareness regarding dyslipidemia, patients were severely undertreated and were unable to reach the target lipid levels while on treatment in Egypt [21, 22]. In addition, there was a lack of appropriate programs for the prevention and timely detection of these two CVD risk factors [23]. Owing to the lack of epidemiologic studies on prevalence or clinical trial data regarding the management of hypertension or dyslipidemia in Egypt, most of the treatment practices were adapted from the evidence-based guidelines of western countries, with modifications based on the cultural, economic, and social lifestyle of the region [24]. Current epidemiology, practices, and overall status of hypertension and dyslipidemia in the local population should advocate incremental reforms in the health care sector, emphasizing on promoting patient awareness, incorporating screening programs, improved treatment, and control of these diseases in Egypt [24–27]. The evaluation of the influence of health care services on patients’ engagement along the common determinants of patient journey touchpoints including awareness, screening, diagnosis, treatment, adherence, and control was recognized as the opportunity to address the unmet needs [28]. However, there is a paucity of evidence-based, high-quality data on these patient-centric outcome measures that would support health care professionals in managing CVDs at the national level. The first step to solve these hindrances is to collect and summarize the existing evidence on the broad research question, which can be done effectively using evidence mapping [29–31].

This semi-systematic review aimed at summarizing the scientific evidence on the prevalence and patient journey stages of hypertension and dyslipidemia in the Egyptian population that would boost policy and practice improvements in the country.

## Main text

### Methods

#### Study design

We conducted a comprehensive semi-systematic data review using structured and unstructured literature searches for retrieval of data on prevalence and various phases of patient-centric management (awareness, screening, diagnosis, treatment, adherence, and control) of hypertension and dyslipidemia in the Egyptian population. The current review adopted the methodological approach described earlier in an associated protocol [32], with minor modifications to address variability in the data or lack of availability of data.

#### Search strategy

We followed a multi-component search strategy. First, a structured search was conducted on the electronic databases MEDLINE and Embase using relevant search strings including Medical Subject Heading (MeSH) terms and their synonyms to identify different phases of patient journey touchpoints for hypertension and dyslipidemia in Egypt. The full search strategy is presented in Supplementary Table 1.

An additional unstructured search was conducted in the Incidence and Prevalence Database (IPD), the WHO and Ministry of Health websites, and Web search engines (the search included a combination of the key MeSH terms from the systematic literature search, with no restrictions on date limits identified in the additional searches).

#### Inclusion and exclusion criteria

The structured search was restricted to systematic reviews and/or meta-analyses, narrative reviews, randomized controlled studies, and observational studies published in the English language from January 1, 2010, to December 31, 2019. Studies with quantitative data on prevalence or at least one component of patient management (awareness, screening, diagnosis, treatment, adherence, and control) involving human adult populations aged ≥18 years were included. Studies involving patient population with hypertension or dyslipidemia, where hypertension was defined as average systolic blood pressure ≥140 mmHg and/or average diastolic blood pressure ≥ 90 mmHg [33] and dyslipidemia was defined as average total cholesterol levels ≥ 5.0 mmol/L or ≥ 190.0 mg/dL [34], were included.

Studies were excluded if they did not focus on hypertension or dyslipidemia or representative of the national population of Egypt. Case studies, letters to the editor, editorials, and studies on special population (pregnant patients, patients with other comorbidities) were also excluded.

#### Study selection

Primary screening and data retrieval were performed by the first independent reviewer on the basis of the titles and abstracts. In the second level of screening, studies were shortlisted by a second independent reviewer on the basis of the pre-defined eligibility criteria and full-text review; any disagreements between the reviewers were resolved through mutual scientific discussions. To account for the unavailability of data on hypertension and dyslipidemia at the national level, additional studies were considered appropriate for inclusion to supplement data collected from structured and unstructured searches. Furthermore, a prominent deviation from the protocol was proposed to adopt a secondary approach, which allowed inclusion of studies conducted on relevant subpopulations (e.g., patients presenting with acute coronary syndrome (ACS)) that could be translated to the real-world setting in the context of prevention and management of these 2 risk factors*.* The shortlisted studies from all sources were analyzed in detail before including in the final records.

#### Data extraction and synthesis

Studies included in the final records were exported to Microsoft Excel after manual screening, followed by retrieval of relevant quantitative data including prevalence of hypertension and dyslipidemia, as well as different phases of patient-centric management (awareness, screening, diagnosis, treatment, adherence, and control). To ensure consistency with real-world experiences, extracted quantitative data were further reviewed and verified.

### Results

#### Screening of studies for hypertension

**Fig. 1:**
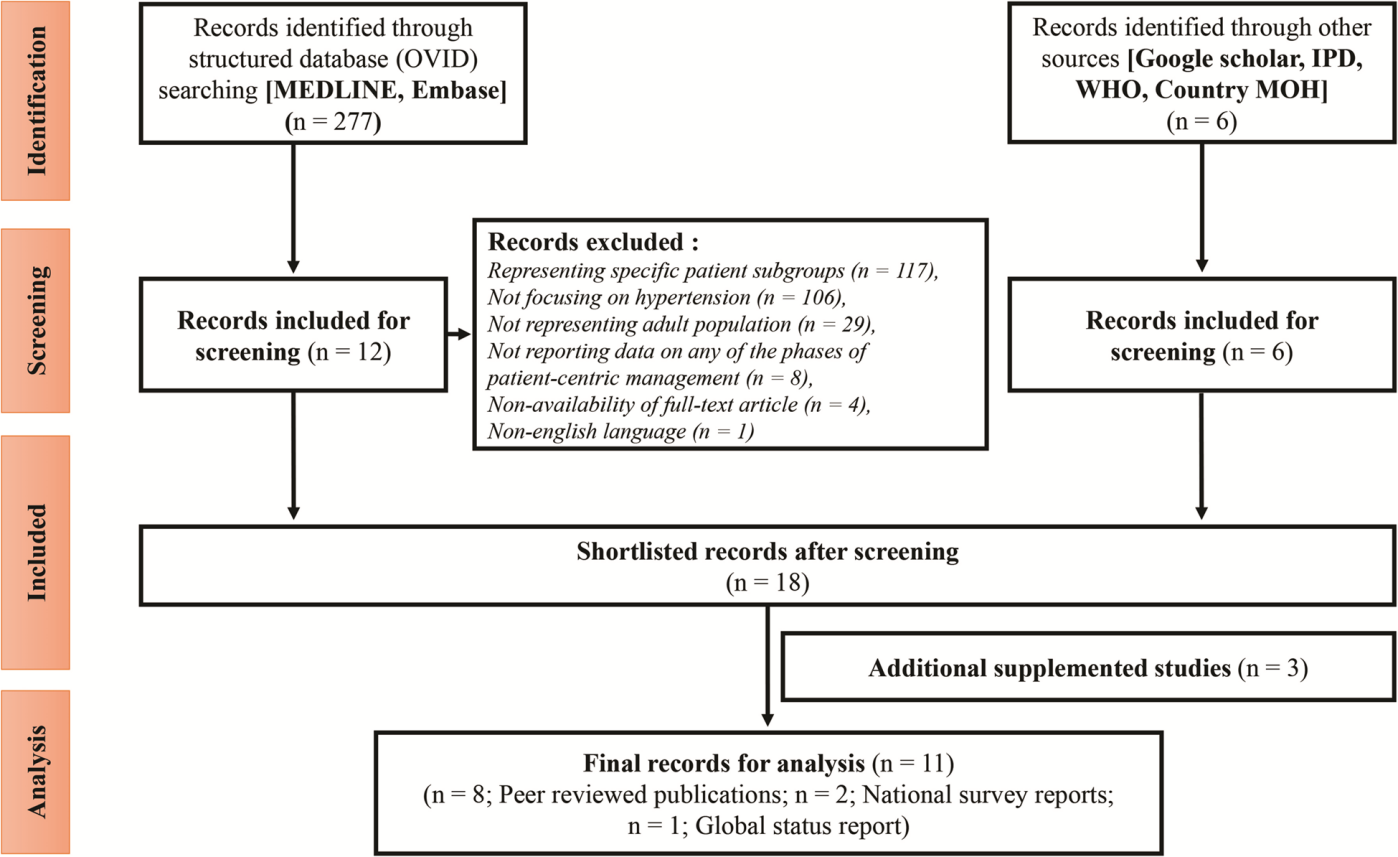
Flowchart of literature search results on hypertension. IPD, Incidence and Prevalence Databases; MOH, Ministry of Health; WHO, World Health Organization

A total of 277 articles from the structured searches and 6 articles from the unstructured searches were retrieved on the prevalence and the distribution of patient journey stages of hypertension. Most of the studies that were excluded represented specific patient subgroups (pregnant women, patients with other comorbidities, *n* = 117). Other studies were excluded for not focusing on hypertension (*n* = 106), not representing adult population (*n* = 29), not reporting data on any of the phases of patient-centric management (*n* = 8), non-availability of full-text article (*n* = 4), and published in a non-English language (*n* = 1). The search results and subsequent data analysis at a later stage allowed inclusion of studies conducted in the subgroup of patients presenting with ACS and coronary artery disease (CAD). Twelve articles from the structured searches and all 6 articles from the unstructured searches were selected for detailed review. Further, 3 additional studies were considered as supplementation to the shortlisted records. Finally, 4 articles from the structured searches [6, 25, 26, 35], 5 studies from the unstructured searches [8, 16, 20, 36, 37], and 2 supplementary studies were included in the final analysis [38, 39]. Ten studies were excluded because of the following reasons: small sample size (*n*=1), meta-analysis (*n*=1), duplicate records (*n*=3), variable definition of hypertension (*n*=1), availability of similar national data (*n*=1), questionnaire-based studies without proper definition of hypertension (*n*=1), anecdotal data (*n*=1), or studies focused on single-pill combination therapy (*n*=1). The literature search and study selection process is summarized in Fig. 1.

#### Description of included studies: hypertension

Three studies including a WHO Global Status Report on NCDs, a nationwide cross-sectional community-based survey, and an Egyptian National Hypertension Project reported prevalence of hypertension ranging from 26 to 29.5% among Egyptian adults [16, 36, 37]. The prevalence of high blood pressure in men and women among the Egypt Health Issues Survey (EHIS) respondents at the time of the survey were 16.7% and 17.2%, respectively [38]. A systematic analysis of a national health examination survey conducted by Ikeda et al. [25] reported hypertension prevalence at 12.1% among individuals aged between 35 and 49 years. In a multicenter, cross-sectional study in Egyptian patients with ACS, the prevalence of hypertension was estimated at 59% [6]. A comparative study between elderly and younger patients with ACS revealed 49.1% incidence of hypertension among the younger population (< 60 years) [35]. In a retrospective study, hypertension was reported in 56.7% of patients with CAD [26].

The data obtained from the most recent EHIS [39] revealed that 43.9% of the Egyptian adult population was aware of their hypertensive condition, whereas only 22.7% of population could achieve blood pressure control. Similarly, awareness about hypertension in the Egyptian National Hypertension Project was estimated at 37.5% [37]. The EHIS showed that 42% of the surveyed population was diagnosed with hypertension [38]. In a systematic review of hypertension in 20 countries, 64.7% of the surveyed population was diagnosed with hypertension, whereas 54.1% received treatment and 39.5% reported control of blood pressure in Egypt [25]. On the basis of the data from Egyptian National Hypertension Project, 24% of hypertensive patients received treatment, whereas only 8% of the patients reported control of blood pressure [37]. A multistage random sampling technique at health insurance clinics in Egypt reported controlled blood pressure in 53.2% of the hypertensive patients and adequate compliance to treatment in 51.9% of the patients [20]. A nationwide Specialized Hypertension Clinics (SHCs) screening and treatment initiative reported that only 27.1% of the hypertensive patients had controlled blood pressure [8]. No study provided data on screening of hypertension in the Egyptian population. The details of the studies included in the final analysis are summarized in Table 1.

**Table 1:** Overview of studies for hypertension included in the final analysis (*N* = 11)

Search type	Study: first author; publication date	Brief study design	Sample size (***N***); characteristics	Prevalence	Awareness	Screening	Diagnosis	Treatment	Adherence	Control	Remarks
**Data as per the MAPS inclusion criteria**											
Unstructured	Global Status Report on noncommunicable diseases: 2014	Global status report tracking worldwide progress in prevention and control of NCDs	Adults (18+ years)	**26.0%**	x	x	x	x	x	x	NA
Supplementary record	Egypt Health Issues Survey: 2015	Cross-sectional survey	*N* = 16,671; individuals (15–59 years) interviewed in the survey.	**17.2% (women)** **16.7% (men)**	x	x	**42.0%**	x	x	x	
Unstructured	Egypt National STEPwise Survey For Noncommunicable Diseases Risk Factors Report; 2017	National cross-sectional community-based household survey	*N* = 6680; nationally representative sample of Egyptian adults (15-69 years).	**29.5%**	x	x	x	x	x	x	
	Registry of the Egyptian specialized hypertension clinics: patient risk profiles and geographical differences: El Faramawy A; 2019	Nationwide Specialized Hypertension Clinics Registry	*N* = 4701; controlled hypertension was defined as BP < 140/90 mmHg in hypertensive patients on antihypertensive medications.	x	x	x	x	x	x	**27.1%**	
**Data deviates from the MAPS inclusion criteria (year limit: 2010–2019)**											
Unstructured	Hypertension Prevalence, Awareness, Treatment, and Control in Egypt. Results from the Egyptian National Hypertension Project (NHP): Ibrahim MM; 1995	Cross-sectional nationwide survey	*N* = 6733; survey participants (25 to 95 years).	**26.3%**	**37.5%**	x	x	**24.0%**	x	**8.0%**	NA
	Patterns and determinants of treatment compliance among hypertensive patients: Youssef RM; 2002	Multistage random sampling technique	*N* = 316; hypertensive patients attending health insurance clinics for prescription refills were randomly selected and interviewed.	x	x	x	x	x	**51.9%**	**53.2%**	
Supplementary record	Prevalence and determinants of hypertension unawareness in Egyptian adults: a cross-sectional study of data from the 2015 Egyptian Health Issues Study: Soliman SS; 2020	Cross-sectional study using data from the 2015 Egyptian Health Issues Survey (EHIS)	*N* = 2869; participants (≥18 years) were included if they measured blood pressure (systolic and/or diastolic pressure ≥ 140/90 mmHg).	x	**43.9%**	x	x	x	x	**22.7%**	
**Data deviates from the MAPS inclusion criteria (definitions/special patient subgroups)**											
Structured	Lipid profile in Egyptian patients with coronary artery disease: Ibrahim MM; 2013	Retrospective study of patients with CAD	*N* = 1000; CAD patients ranged from 19 to 90 years.	**56.7%**	x	x	x	x	x	x	Data extracted from patients with stable CAD or had a history of MI
	Control of hypertension with medication: a comparative analysis of national surveys in 20 countries: Ikeda N; 2014	Systematic search of national health examination surveys from 20 countries	*N* = 3342; Individuals (35–49 years).	**12.1%**	x	x	**64.7%**	**54.1%**	x	**39.5%**	Hypertension definition is based on Systolic blood pressure (i.e., < 140 mmHg)
	Comparative study between elderly and younger patients with acute coronary syndrome: Obaya M; 2015	Retrospective registry	*N* = 570; ACS patients were divided into 2 groups: elderly ≥ 60 years; younger < 60 years.	**49.1**	x	x	x	x	x	x	Data extracted from younger patients (< 60 years) presenting with ACS; hypertension not defined
	The pattern of risk-factor profile in Egyptian patients with acute coronary syndrome: phase II of the Egyptian cross-sectional CardioRisk project: Reda A; 2019	Multi-center, observational, cross-sectional study of ACS patients	*N* = 1681; ACS patients (≥18 years) having a history of hypertension or blood pressure (systolic and/or diastolic pressure ≥140/90 mmHg).	**59.0%**	x	x	x	x	x	x	Data extracted on investigating ACS patients

*ACS*, acute coronary syndrome; *CAD*, coronary artery disease; *MI*, myocardial infarction; *NCD*, noncommunicable diseases; *NA*, not applicable

#### Screening of studies for dyslipidemia

**Fig. 2:**
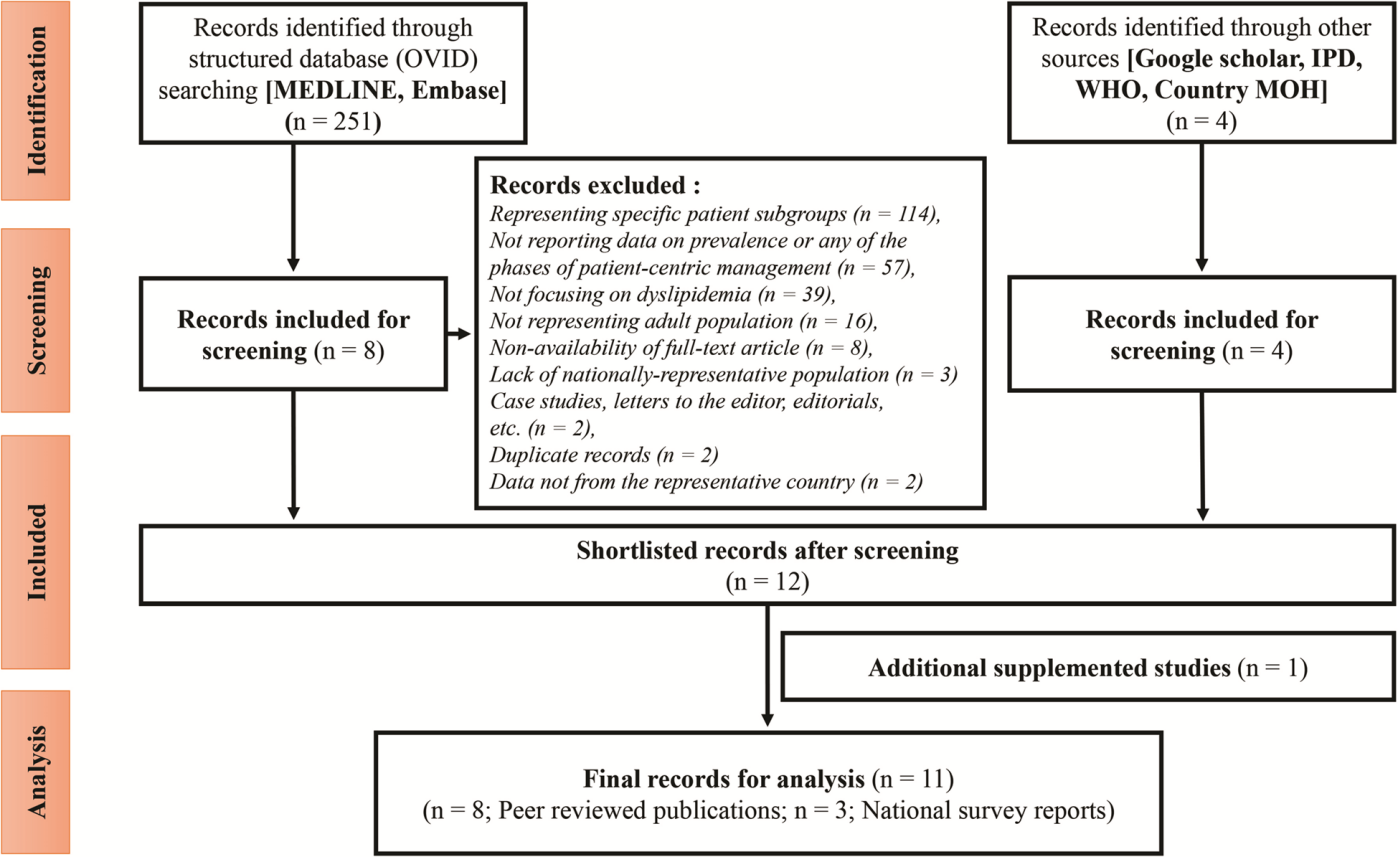
Flowchart of literature search results on dyslipidemia. IPD, Incidence and Prevalence Databases; MOH, Ministry of Health; WHO, World Health Organization

A total of 251 articles from the structured searches and 4 articles from the unstructured searches were retrieved on prevalence and patient-centric management of dyslipidemia for the Egyptian population. Studies representing specific patient subgroups (pregnant women, patients with other comorbidities, *n* = 114), not reporting data on prevalence or any of the phases of patient-centric management (*n* = 57), not focusing on dyslipidemia (*n* = 39), not representing adult population (*n* = 16), non-availability of full-text article (*n* = 8), lack of nationally-representative population (*n* = 3), case studies, letters to the editor, editorials (*n* = 2), duplicate records (*n* = 2), and data not from the representative country (*n*=2) were excluded. The findings of the initial data review indicated lack of data on dyslipidemia in the general population and thus necessitated inclusion of patient subgroups diagnosed with ACS or CAD at a later stage. Eight articles from the structured searches, and all 4 articles from the unstructured searches were considered eligible for review. An additional survey-based study on NCDs risk factors in Egypt was supplemented to the shortlisted records for assessment. Finally, 8 articles from the structured searches [6, 8, 21–23, 26, 35, 40], 2 nationwide survey reports from the unstructured search [41, 42], and 1 additional study was included in the final analysis [16]. Two of the selected studies were not considered for final analysis because of the availability of the similar data in the STEPS Survey reports. The literature search and study selection process are summarized in Fig. 2.

#### Description of included studies: dyslipidemia

On the basis of the nationwide survey data, the prevalence of hypercholesterolemia was 19.4%, 36.8%, and 19.2% in the Egyptian population during 2006, 2012, and 2017, respectively [16, 41, 42]. However, cholesterol screening was poor, as reported in one nationwide survey (8.6%) [16]. A nationwide, cross-sectional CardioRisk study conducted in patients with ACS reported increased low-density lipoprotein cholesterol (LDL-C) levels in 52.5% of the patients [6]. Similarly, another retrospective study reported a prevalence of dyslipidemia in 50.9% of younger patients (< 60 years) diagnosed with ACS [35]. The high prevalence of dyslipidemia (58.7%) based on total cholesterol levels was also identified in Egyptian patients with CAD [26]. However, in a group of hypertensive Egyptians attending SHCs, the prevalence of dyslipidemia was only 8.9% [8].

The results from both the Centralized Pan-Middle East Survey on the under-treatment of hypercholesterolemia (CEPHEUS) studies in Egypt indicated that most patients were aware of their target cholesterol levels (76%) or bad cholesterol (75%) [23, 40]. Although compliance to treatment of dyslipidemia in the CEPHEUS II study was reported at 59% [40], target LDL-C levels were achieved in only 32.5% and 34.4% of the patients as reported in the CEPHEUS I and CEPHEUS II results, respectively [23, 40]. According to the DYSlipidemia International Study (DYSIS)-Egypt, one-third of the patients who received chronic statin treatment had control in LDL-C levels [21]. In the DYSIS II study, 73.9% of patients with ACS received treatment for dyslipidemia, whereas the proportion of patients who achieved target LDL-C levels varied within different risk categories, with 5.1% at very high risk, 27.3% at high risk, 32.3% at moderate risk, and 14.3% at low risk [22]. No study provided data on diagnosis of dyslipidemia in the Egyptian population. The details of the studies included in the final analysis are summarized in Table 2.

**Table 2:** Overview of studies for dyslipidemia included in the final analysis (*N* = 11)

Search type	Study: first author; publication date	Brief study design	Sample size (***N***); characteristics	Prevalence	Awareness	Screening	Diagnosis	Treatment	Adherence	Control	Remarks
**Data as per the MAPS inclusion criteria**											
Unstructured	Ministry of Health & population, Egypt Preventive Sector Central Epidemiology and Disease Surveillance (ESU) Non-Communicable Disease Surveillance Unit (NCDSU); 2006	National cross-sectional survey	*N* = 9780; participants (15–65 years)	**19.4%**	x	x	x	x	x	x	NA
	WHO and ARE-Ministry of Health & Population: Egypt National STEPwise Survey of Non-Communicable Diseases Risk Factors 2011-2012; 2012	Multistage cluster sample design	*N* = 5300; participants (15–65 years)	**36.8%**	x	x	x	x	x	x	NA
Supplementary Record	Egypt National STEPwise Survey For Noncommunicable Diseases Risk Factors Report; 2017	National cross-sectional community-based household survey	*N* = 6680; nationally representative sample of Egyptian adults (15–69 years).	**19.2%**	x	**8.6%**	x	x	x	x	NA
**Data deviates from the MAPS inclusion criteria (definitions/special patient subgroups)**											
Structured	The DYSlipidemia International Study (DYSIS)-Egypt: A report on the prevalence of lipid abnormalities in Egyptian patients on chronic statin treatment: El Etriby A; 2013	Cross-sectional, observational, multinational study	*N* = 1458; patients (≥ 45 years) on stable statin treatment.	x	x	x	x	x	x	**33.7%**	Control data extracted based on target LDL-C attainment
	Lipid profile in Egyptian patients with coronary artery disease: Ibrahim MM; 2013	Retrospective consecutive sampling of patients with CAD	*N* = 1000; patients ranged from 19 to 90 years.	**58.7%**	x	x	x	x	x	x	Data extracted from patients with stable CAD or had a history MI
	Centralized Pan-Middle East Survey on the Under- Treatment of Hypercholesterolemia: Results from the CEPHEUS Study in Egypt: Reda A; 2014	Multicenter, observational study	*N* = 1043; subjects (≥ 18 years) were receiving lipid-lowering drug treatment.	x	**76.0%**	x	x	x	x	**32.5%**	Control data extracted based on target LDL-C attainment
	Comparative study between elderly and younger patients with acute coronary syndrome: Obaya M; 2015	Comparative study between elderly and younger patients with ACS	*N* = 570; patients were divided into 2 groups: elderly ≥ 60 years; younger < 60 years.	**50.9**	x	x	x	x	x	x	Data extracted from younger patients (< 60 year) presenting with ACS; dyslipidemia not defined
	Centralized Pan-Middle East Survey on the Under- Treatment of Hypercholesterolemia: Results from the CEPHEUS II Study in Egypt: Reda A; 2017	Observational, multicenter, cross-sectional survey	*N* = 896; Subjects (≥ 18 years) receiving lipid-lowering drug treatment.	x	**75.0%**	x	x	x	**59.0%**	**34.4%**	Data extracted based on LDL-C levels
	Prevalence of lipid abnormalities and cholesterol target value attainment in Egyptian patients presenting with an acute coronary syndrome: Sobhy M; 2018	Prospective, observational study of patients presenting with ACS	*N* = 199; patients (≥ 18 years) who were hospitalized for ACS and receiving lipid-lowering treatment.	x	x	x	x	**73.9%**	x	**5.1%** (very high risk; < 70 mg/dL) **27.3%** (high risk; < 100 mg/dL) **32. 3%** (moderate risk; < 115 mg/dL) **14.3%** (low risk; < 130 mg/dL)	Data extracted on investigating selected subpopulations (ACS patients); control data extracted based on target LDL-C attainment corresponding to different risk categories
	The pattern of risk-factor profile in Egyptian patients with acute coronary syndrome: phase II of the Egyptian cross-sectional CardioRisk project: Reda A; 2019	Multi-center, observational, cross-sectional study of patients presenting with ACS	*N* = 1681; participants (≥18 years) having a history of lipid-lowering therapy or LDL-C > 70 mg/dL (> 1.81 mmol/L).	**52.50%**	x	x	x	x	x	x	Data extracted on investigating ACS patients; dyslipidemia definition is based on LDL-C levels
	Registry of the Egyptian specialized hypertension clinics: patient risk profiles and geographical differences: El Faramawy A; 2019	Nationwide Specialized Hypertension Clinics Registry	*N* = 4701; dyslipidemia considered when LDL ≥ 130 mg/dL, HDL ≤ 50 mg/dL in women and ≤ 40 mg/dL in men, and TG ≥150 mg/dL or if the patient was receiving a lipid-lowering agent	**8.90%**	x	x	x	x	x	x	Data extracted on investigating hypertensive patients based on LDL-C, HDL, and TG levels

*ACS*, acute coronary syndrome; *HDL*, high-density lipoprotein; *CAD*, coronary artery disease; *LDL-C*, low-density lipoprotein cholesterol; *MI*, myocardial infarction; *NA*, not applicable; *TG*, triglycerides

### Discussion

This semi-systematic review, for the first time, presented the evidence available and identified inconsistencies in reporting the data on prevalence and patient journey stages (awareness, screening, diagnosis, treatment, adherence, and control) of hypertension and dyslipidemia in Egypt. Despite performing a comprehensive literature review, only 22 studies (11 studies each for hypertension and dyslipidemia) were found to be relevant for the final analysis. Data were available on prevalence and all phases of patient-centric management of hypertension, except screening rates, whereas data were available on the prevalence and all phases of patient-centric management of dyslipidemia, except diagnosis rates.

#### Comparison with other studies on hypertension

The studies included in this review demonstrated a wide variability in the prevalence of hypertension, which could be explained by differences in population characteristics, subgroup analysis, and study methodologies. The prevalence estimates were lower in the younger general population aged between 35 and 49 years (12%) [25] and surveyed population aged < 60 years (17%) [38], whereas the estimates were higher ranging from 49 to 59% in subgroup studies of patients with ACS and CAD, indicating hypertensive patients were more susceptible to develop CVDs [6, 26, 35]. However, the prevalence of hypertension in the general Egyptian population did not vary and ranges between 26 and 29.5% [16, 36, 37], which were comparable to the prevalence reported in the LMICs (31%) [43] and the USA (29%) [44].

Our study demonstrated that the levels of awareness of hypertension (38% and 44%) [37, 39] in the Egyptian population were comparable with those in LMICs (38%), but markedly lower when compared with that in the high-income countries (HICs) (67%) [45]. Consequently, the percentages of diagnosed hypertensive individuals in Egypt were 42% and 65% in 2 studies [25, 38], which were comparable to the range reported for various LMICs (46–75%), but substantially lower compared to the USA (85%) [25]. Further, we observed a wide variability in the proportion of patients treated for hypertension (24% and 54%) [25, 37], which was not consistent with the treatment rates reported for LMICs (29%) and HICs (56%) [45]. In our review, patient compliance to treatment for hypertension was reported at 52%, which was higher than the patient compliance reported in a recent study (41%) [46]. Lower compliance to anti-hypertensive medication could be attributed to increased cost, side effects, monotherapy, and lack of efficacy [20]. Similarly, there is a wide variability in the blood pressure control rates as reported in our review ranging from as the lowest estimate of 8% to the highest estimate of 53% [8, 20, 25, 37, 39], whereas the control rates were reported at 27% in LMIC and 51% in HICs [45]. This trend in the rates of awareness, treatment, adherence, and control of hypertension in the Egyptian population that mimicked the trends in LMICs might be related to lack of compliance with evidence-based treatment, unavailability of simplified recommendations, and higher diagnostic thresholds limiting early detection and treatment initiation [43, 47, 48].

#### Comparison with other studies on dyslipidemia

According to the STEPS reports, the prevalence of dyslipidemia in the general population of Egypt fluctuated from 19.4% in 2006 to 36.8% in 2012 and reduced to 19.2% in 2017 [16, 41, 42]. Although reasons for wide variability in prevalence could not be identified, such evidence will be useful for future endeavors in planning large-scale studies in implementing treatment interventions or health care policies. Among Egyptian patients with ACS and CAD, the prevalence of dyslipidemia was consistently higher within the range between 51 and 59%, which reinforced the latter’s status as a major risk factor in CVDs [6]. There was a correlation between the 2 risk factors when a study in hypertensive patients reported prevalence of dyslipidemia in 9% of the patients [8]. It is imperative to identify such high-risk patients with the presence of multiple risk factors so that they can be targeted for treatment to reduce CVDs. The prevalence of dyslipidemia reported in the general Egyptian population was much lower than that projected by the WHO (39%). Further, the prevalence of high cholesterol in Egypt was comparatively lower than that reported in the USA (55%) and UK (66%) [15]. Data from both the CEPHEUS studies indicated a higher level of awareness in patients about their cholesterol levels (≥75%), with a moderate level of adherence to the therapeutic regimen (59%) [23, 40]. However, there was a possibility of recall bias, which is commonly seen in survey studies. Similarly, the proportion of patients treated (74%) was relatively higher when analyzed in a small cohort of patients suffering from ACS [22], which may not be representative of the overall Egyptian population.

Although the treatment rates of dyslipidemia were higher, the overall control rates were lower in the general population as well as in patients with ACS, ranging from 20 to 34% [21–23, 40]. These lower control rates could be attributed to a lack of implementation of evidence-based guidelines, clinical inertia, and patients’ non-compliance to the prescribed treatment [49]. Similarly, less affordability to costly medications and “ceiling effect” on LDL-C lowering by statin therapy could be the reason for poor control rates in the lipid levels [50, 51].

#### Implications for practice and patient-centric recommendations

Suboptimal control of hypertension reflected lack of education and limited medical access in Egypt, indicating the need for collaborative efforts from health authorities, medical community, and pharma companies to promote patient education and national level campaigns [19, 52]. Further, there is a need for specialized hypertension clinics in Egypt to improve quality and accessibility to health care services for the patients [11]. Egypt is now steadily progressing with its recent initiative to develop a nationwide screening program “100 Million Healthy Lives” accompanied by awareness and treatment campaigns for NCDs such as hypertension and diabetes” [53]. Thus, it would be crucial to publish these reliable and meaningful findings allowing the use of the credible information to aid policymakers to identify the target population for their interventions and policies. Moreover, extending the campaign to dyslipidemia would impart a comprehensive coverage of the CVD landscape in Egypt. In addition to these measures, there is a need to conduct large epidemiological studies encompassing cultural and ethnic contexts and studies comparing evidence-based treatment and current treatment approaches in Egypt to improve the rates of awareness, treatment, and control of dyslipidemia [54, 55]. Similarly, awareness promotion, early identification, timely treatment, and cost-effective management of hypertension and dyslipidemia could be achieved by an integrated approach between affordable primary health care (PHC) services and family- or community-level focus [56–60], considering the medical resources constraint in Egypt. Although there were significant advances in the emergency management of CVDs, primary preventive care was offered by the general practitioners (GPs), whereas specialists focused on secondary preventive measures for CVDs. Owing to a lack of formal education on cardiac primary prevention, there is a need for restructuring curriculum in cardiology training with more focus on primary preventive measures [5]. Moreover, physician’s lack of familiarity with diagnostic guidelines and practice recommendations indicated that there is a need to facilitate workshops, seminars, and availability of international manuals for PHCs [61]. Although the WHO has recognized Egypt’s commitment in scaling up PHC facilities [62], the imbalance in care services across different regions and socioeconomic groups necessitated the adoption of comprehensive strategies leading to effective national health care reforms [63]. To achieve these reforms, the overall burden of hypertension and dyslipidemia in the Egyptian population should be estimated using a risk assessment approach. In the absence of recommendations for opportunistic screening for CVD risk assessment in the general population, the European guidelines emphasized the importance of systematic screening for CVD risk assessment in individuals who are at high risk due to positive family history for CVD risk factors [64]. Such an approach in addition to the periodic data collection to estimate the precise prevalence of risk factors and integration of population-based surveillance programs into the national health information systems targeted toward detection, treatment, and control of CVDs could be rigorously followed as proposed in the WHO’s Global Action Plan [65]. For decades, Egypt’s health care sector has struggled to address the public health crisis owing to underinvestment in public hospitals, unreliable private health care, inadequate health insurance, and issues related to equity by race/ethnicity, gender, and other socioeconomic status measured by education, income, or occupation [66, 67]. However, recently the Egyptian government has prioritized health care services for all citizens ensuring mandatory coverage eliminating existing disparities through “The Universal Health Coverage Project” [68].

In Egypt, barriers were identified toward execution of smoking prevention measures such as lack of enforcement of smoke-free laws and lack of penalties for violators [69]. National health policies and community programs targeting the concurrent risk factors such as smoking cessation, improvement in lifestyle, and innovations within the context of patient-centric care, increasing health communications, and enabling treatment adherence monitoring could be meaningful measures for hypertension and cholesterol management [70]. The implementation of an electronic monitoring system and the use of eHealth communication were found to be complicated and seemed to be underused [71]. Despite the designing of several eHealth applications, successful implementation remained a challenge in Egypt due to lack of patient acceptance, financial constraints, and limited infrastructure, especially in the rural areas [72]. However, Egypt’s approach to digitize health services has witnessed an accelerated transformation with the emerging use of artificial intelligence to diagnose diseases and provide treatment solutions via telehealth and telemedicine, enabling better handling of medical data during the COVID-19 pandemic [73]. This holistic approach of translating digital interventions to the most vulnerable patients with greater CVD risk factors can be adopted into the National eHealth policy as well in the future.

### Limitations

Our review has some limitations. Data on key patient journey touchpoints were not consistently available in all articles, and thus, pooled estimates of synthesized data could not be reported. Furthermore, case studies and studies conducted on some patient subgroups (e.g., pregnant women, adolescents) were excluded, and therefore, we might have missed evidence to describe some of the observations in our review. In addition, exclusion of patients with other comorbidities especially diabetes might have underestimated the actual prevalence of hypertension because of the increased occurrence of hypertension in patients with diabetes. Additionally, this study is limited by inclusion of dyslipidemia studies based only on the levels of total cholesterol and not considering other variables such as triglycerides, low-density lipoprotein, and high-density lipoprotein. It is important to note that evidence maps can provide a brief overview of topic; however, no information on efficacy of any individual treatment intervention was provided for patients with hypertension or dyslipidemia. Furthermore, in this semi-systematic review of articles, critical appraisal for the quality of findings was not assessed in the included studies.

## Conclusion

This study complemented the existing literature on prevalence and provided insights on different phases of patient-centric management of hypertension and dyslipidemia in Egypt. The findings supported the need for opportunistic screening for CVD risk factors when visiting a PHC facility, which in turn could result in early diagnosis and improve treatment outcomes. The need for multi-disciplinary commitment from government, policymakers, health care professionals, and other stakeholders toward the prevention of CVD risk factors, promotion of lifestyle interventions, and overall disease management systems is crucial. Finally, this study may provide a basis for research prioritization and recommendations and guidance to practice and amend health policies for the management of hypertension and dyslipidemia in Egypt. The current study reinforces the need to generate more high-quality data at national-level on prevalence of hypertension and dyslipidemia along with patient journey touchpoints to validate the conclusion of our findings.

## Supplementary Information

Additional file 1: Table S1. Search Strategy for Structured Search

## Data Availability

The dataset generated and analyzed during the current study is not publicly available as the dataset is proprietary, but is available from the corresponding author on reasonable request.

## Abbreviations

ACS: Acute coronary syndrome
CVD: Cardiovascular disease
CEPHEUS: Centralized Pan-Middle East Survey on the under-treatment of hypercholesterolemia
CAD: Coronary artery disease
DYSIS: DYSlipidemia International Study
EHIS: Egypt Health Issues Survey
GP: General practitioner
HICs: High-income country
IPD: Incidence and Prevalence Database
LMIC: Low- and middle-income country
LDL-C: Low-density lipoprotein cholesterol
MeSH: Medical Subject Heading
NCDs: Non-communicable diseases
PHC: Primary health care
SHC: Specialized hypertension clinic
SBP: Systolic blood pressure
WHO: World Health Organization
