# Supplementary material for: A semi-systematic review on hypertension and dyslipidemia care in Egypt—highlighting evidence gaps and recommendations for better patient outcomes
Source: J Egypt Public Health Assoc. 2021 Dec 1;96:32. doi: 10.1186/s42506-021-00096-9 (PMC8634749; doi:10.1186/s42506-021-00096-9)
Supplement: Supplementary file 1 — Table S1. Search Strategy for Structured Search [file 42506_2021_96_MOESM1_ESM.docx]

**Supplementary Table 1**. Search Strategy for Structured Search

| **Hypertension** | | | |
| --- | --- | --- | --- |
| **Database Searched** | **Keywords** | **Limits Applied** | **Exclusion Criteria for Screening** |
| MEDLINE, Embase | (hypertension **OR** blood pressure **OR** hypertensives)  **AND**  (epidemiology **OR** prevalence **OR** incidence **OR** national **OR** survey **OR** registry)  **AND**  (awareness **OR** knowledge **OR** health literacy **OR** screening **OR** diagnosis **OR** diagnosed **OR** undiagnosed **OR** treatment **OR** treated **OR** untreated **OR** control **OR** controlled **OR** uncontrolled **OR** adherence **OR** compliance **OR** adhere **OR** therapy **OR** non-adherence)  **AND**  Egypt | 1. Time period: January 1, 2010 to December 31, 2019 2. Language: English 3. Species: Humans, Human | 1. <18 years of age 2. Not hypertension 3. Relevant patient journey data NA 4. Full text NA 5. Specific patient subgroups such as patients with comorbidities, pregnant women 6. Not English language 7. Case studies, letter to editors, editorials 8. Duplicate records 9. Data lacking national representativeness 10. Data not from representative country |
| **Dyslipidemia** | | | |
| MEDLINE, Embase | (dyslipidemia **OR** hypercholesterolemia **OR** cholesterol **OR** triglycerides **OR** LDL)  **AND**  (epidemiology **OR** prevalence **OR** incidence **OR** national **OR** survey **OR** registry **OR** Statistics)  **AND**  (health literacy **OR** screening **OR** awareness **OR** knowledge **OR** treated **OR** treatment **OR** diagnosis **OR** undiagnosed **OR** diagnosed **OR** therapy **OR** controlled **OR** control **OR** uncontrolled **OR** adherence **OR** adhere **OR** compliance)  **AND**  United Arab Emirates **OR** Algeria **OR** South Africa **OR** Africa **OR** Egypt **OR** Saudi Arabia **OR** Middle East | 1. Time period: January 1, 2010 to December 10, 2019 2. Language: English 3. Species: Humans, Human | 1. <18 years of age 2. Not dyslipidemia 3. Relevant patient journey data NA 4. Full text NA 5. Specific patient subgroups such as patients with comorbidities, pregnant women 6. Not English language 7. Case studies, letter to editors, editorials 8. Duplicate records 9. Data lacking national representativeness 10. Data not from representative country |
| LDL, low-density lipoprotein; NA, not available. | | | |
